# The Use of Green Coffee Extract as a Weight Loss Supplement: A Systematic Review and Meta-Analysis of Randomised Clinical Trials

**DOI:** 10.1155/2011/382852

**Published:** 2010-08-31

**Authors:** Igho Onakpoya, Rohini Terry, Edzard Ernst

**Affiliations:** Complementary Medicine, Peninsula Medical School, University of Exeter, 25 Victoria Park Road, Exeter EX2 4NT, UK

## Abstract

The purpose of this paper is to assess the efficacy of green coffee extract (GCE) as a weight loss supplement, using data from human clinical trials. Electronic and nonelectronic searches were conducted to identify relevant articles, with no restrictions in time or language. Two independent reviewers extracted the data and assessed the methodological quality of included studies. Five eligible trials were identified, and three of these were included. All studies were associated with a high risk of bias. The meta-analytic result reveals a significant difference in body weight in GCE compared with placebo (mean difference: −2.47 kg; 95%CI: −4.23, −0.72). The magnitude of the effect is moderate, and there is significant heterogeneity amongst the studies. It is concluded that the results from these trials are promising, but the studies are all of poor methodological quality. More rigorous trials are needed to assess the usefulness of GCE as a weight loss tool.

## 1. Introduction

Overweight and obesity have become a serious health concern [1]. Different weight management strategies are presently utilised, and a variety of weight loss supplements sold as “slimming aids” are readily available. However, the efficacy of some of these food supplements remains uncertain. One such supplement is the green coffee extract (GCE). 

GCE is present in green or raw coffee [2]. It is also present in roasted coffee, but much of the GCE is destroyed during the roasting process. Some GCE constituents, such as chlorogenic acid (CGA) can also be found in a variety of fruits and vegetables [3]. The daily intake of CGA in persons drinking coffee varies from 0.5 to 1 g [4]. The traditional method of extraction of GCE from green coffee bean, *Coffea canephora robusta*, involves the use of alcohol as a solvent [5]. Extracted GCE is marketed as a weight loss supplement under a variety of brand names as a weight loss supplement such as “Coffee Slender”, and “Svetol”. 

Evidence is accumulating from animal studies regarding the use of GCE as a weight loss supplement [6, 7]. In human subjects, coffee intake has been reported to be inversely associated with weight gain [8]. Consumption of coffee has also been shown to produce changes in several glycaemic markers in older adults [9]. Similarly, other research has indicated that the consumption of caffeinated coffee can lead to some reductions in long-term weight gain, an effect which is likely to be due to the known thermogenic effects of caffeine intake as well as effects of GCE and other pharmacologically active substances present in coffee [10]. GCE has also been postulated to modify hormone secretion and glucose tolerance in humans [11]. This effect is accomplished by facilitating the absorption of glucose from the distal, rather than the proximal part of the gastrointestinal tract.

The objective of this paper is to analyse the results of human clinical trials assessing the efficacy of GCE as a weight-reducing agent.

## 2. Methods

Electronic searches of the literature were conducted for the following databases: MEDLINE, EMBASE, CINAHL, AMED, and *The Cochrane Library*. Each database was searched from inception up until April, 2010. Search terms used included coffee, green coffee, green coffee extract, roasted coffee, decaffeinated coffee, chlorogenic acid, caffeoylquinic acid, antiobesity agent, appetite suppressant, abdominal fat, BMI, body mass index, body fat, body weight, overweight, over weight, corpulen*, obes*, weight loss, weight decrease, weight watch, weight cycle, weight control, weight gain, weight maintenance, weight reduction, weight change, dietary supplement, food supplement, nutraceutical, nutri*supplement, over-the-counter OR OTC, nonprescription drugs, randomised controlled trial, clinical trial, and placebo. We also searched other internet databases for relevant conference proceedings, as well as our own files. Hand searches of the bibliography of retrieved full texts were also conducted.

Only randomised, double-blind, and placebo-controlled studies were included in this paper. To be considered for inclusion, studies had to test the efficacy of GCE for weight reduction in obese or overweight humans. Included studies also had to report body weight and/or body mass index (BMI) as an outcome. No age, time, or language restrictions were imposed for inclusion of studies. Studies which involved the use of GCE as part of a combination treatment or not involving obese or overweight subjects were excluded from this paper. 

Two independent reviewers assessed the eligibility of studies to be included in the paper. Data were extracted systematically by two independent reviewers according to the patient characteristics, interventions, and results. The methodological quality of all included studies was assessed by the use of a quality assessment checklist adapted from the consolidated standard of reporting trials (CONSORT) guidelines [12, 13]. Disagreements were resolved through discussion with the third author.

Data are presented as means with standard deviations. Mean changes in body weight were used as common endpoints to assess the differences between GCE and placebo groups. Using the standard meta-analysis software [14], we calculated mean differences (MD) and 95% confidence intervals (CI). The *I* ^2^ statistic was used to assess for statistical heterogeneity amongst studies.

## 3. Results

**Figure 1:**
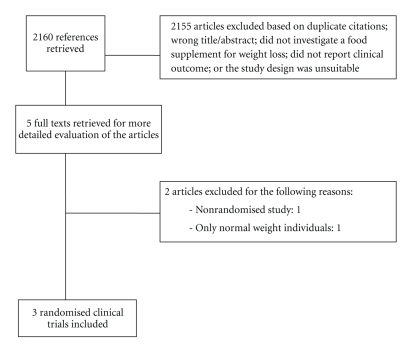
Flow chart for inclusion of randomised clinical trials.

Our searches produced 2160 “hits”. 328 articles were excluded because they were duplicate citations, while 767 articles were excluded because of wrong titles and abstracts. Another 598 articles were excluded because they did not investigate a food supplements, and 454 articles excluded due to no report on clinical outcome. A further 13 articles were excluded due to unsuitable study design. Thus, 5 potentially relevant articles were identified (Figure 1). One trial was excluded because it involved only normal weight individuals, and did not measure weight as an outcome [15]. Another trial was excluded because it was not randomised [16]. In effect, 3 randomised clinical trials (RCTs) including a total of 142 participants met our inclusion criteria, and were included in this systematic paper [5, 17, 18]. Their key details are summarized in Tables 1 and 2.

**Figure 2:**
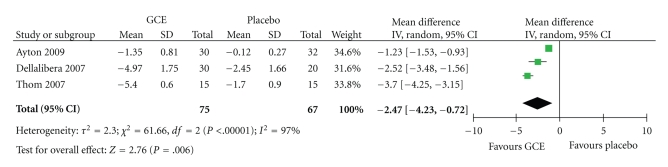
Forest plot showing the effect of GCE on body weight.

A forest plot (random-effect model) for the three trials is shown in figure 2. The meta-analysis reveals a statistically significant difference in body weight between GCE and placebo (MD: −2.47 kg; 95% CI: −4.23, −0.72). The *I* ^2^ statistic of 97% suggests that there is considerable heterogeneity amongst the studies. A further plot of two trials which involved CGA-enriched GCE revealed a statistically nonsignificant difference in body weight between GCE and placebo (MD: −1.92 kg; 95% CI: −5.40, 1.56). Heterogeneity was also considerable in this analysis (*I* ^2^ statistic of 99%). One of the studies reported a statistically significant decrease in the percentage of body fat in the GCE group compared with baseline, but no significant difference in the placebo group [5]. There was no mention of intergroup differences regarding the percentage of body fat. None of the trials reported any adverse events associated with the use of GCE.

## 4. Discussion

The main purpose of this systematic paper was to assess the efficacy of GCE as a weight loss supplement. The overall meta-analysis revealed a significant difference in change in body weight between GCE and placebo. The magnitude of this significance is moderate, and the clinical relevance is therefore not certain. There is also considerable heterogeneity amongst the three trials.

In animals, GCE has been reported to influence postprandrial glucose concentration and blood lipid concentration [5]. This is thought to be via reduction in the absorption of glucose in the intestine; a mechanism achieved by promoting dispersal of the Na^+^ electrochemical gradient. This dispersal leads to an influx of glucose into the enterocytes [19]. GCE is also thought to inhibit the enzymatic activity of hepatic glucose-6-phosphatase, which is involved in the homeostasis of glucose [20]. Reports from animal studies have suggested that GCE mediates its antiobesity effect possibly by suppressing the accumulation of hepatic triglycerides [6]. Some authors have also posited that the antiobesity effect of GCE may be mediated via alteration of plasma adipokine level and body fat distribution and downregulating fatty acid and cholesterol biosynthesis, whereas upregulating fatty acid oxidation and peroxisome proliferator-activated receptor alpha (PPAR*α*) expression in the liver [7]. 

Diets rich in polyphenols may help to prevent various kinds of diseases associated with oxidative stress, including coronary heart disease and some forms of cancer [21, 22]. GCE has been reported to have antioxidant activity, demonstrated by its ability to scavenge free radicals *in vitro*, and to increase the antioxidant capacity of plasma *in vivo* [16, 23]. There is also evidence that certain dietary phenols, including GCE, may modify intestinal glucose uptake in a number of ways [8, 24]. This activity might provide a basis for explaining its effects on body weight. The purported slimming effect of GCE would have a protective effect against diabetes mellitus, via changes in gastrointestinal hormone secretion [10]. A few questions, however, arise from the RCTs which involve the use of GCE as a weight loss aid. 

All the RCTs involving the use of GCE which have been conducted so far have very small sample sizes, with the largest number of participants being 62 in one trial [17]. These small sample sizes increase the possibility of spurious or false positive results. Two of the RCTs were unclear about drop-outs of participants from the trial; neither did they report on intention-to-treat analysis [17, 18]. All of the trials so far identified have been of very short duration. This makes it difficult to assess the efficacy and safety of GCE as a weight reduction agent on the medium to long-term. Although none of the RCTs identified reported any adverse events, this does not indicate that GCE intake is “risk-free”. Two participants in a study report dropped out due to adverse events associated with the intake of GCE [16]. These included headache and urinary tract infection. Thus, the safety of this weight loss aid is not established.

The effective dosage of GCE for use as a weight loss supplement is also not established. The dosages of GCE reported in most of the human trials identified were estimated, as the GCE was a component of coffee. While 2 of the RCTs identified enriched their GCE with CGA [5, 17], the third trial did not report that the GCE used was fortified with CGA [18]. This warrants further investigation.

The RCTs identified from our searches were not also clear on blinding issues. None of the RCTs reported on how randomisation was carried out, and none provided information regarding blinding of outcome assessors. This casts doubt on the internal validity of these trials. Future trials involving the use of GCE as a weight loss supplement should be conducted in line with the CONSORT guidelines. This will ensure the validity and applicability of study results. Two authors in one study were affiliated to a company which markets Svetol [18] but did not specify whether or not they had any conflicts of interest. 

This systematic review has several limitations. Though our search strategy involved both electronic and nonelectronic studies, we may not have identified all the available trials involving the use of GCE as a weight loss supplement. Furthermore, the methodological quality of the studies identified from our searches is poor, and all are of short duration. These factors prevent us from drawing firm conclusions about the effects of GCE on body weight.

## 5. Conclusion

The evidence from RCTs seems to indicate that the intake of GCE can promote weight loss. However, several caveats exist. The size of the effect is small, and the clinical relevance of this effect is uncertain. More rigorous trials with longer duration are needed to assess the efficacy and safety of GCE as a weight loss supplement.

## Figures and Tables

**Table 1:** Methodological characteristics of included studies.

Author Year Country	Main outcome (s)	Main diagnoses of study participants	Study design	Gender M/F	Randomisation appropriate^?^	Allocation concealed^?^	Groups similar at baseline^?^	Similar follow-up of groups^?^	Outcome assessor blinded^?^	Care provider blinded^?^	Patients blinded^?^	Attrition bias^?^	ITT analysis^?^
*Ayton Research 2009 United Kingdom	Body weight, waist, bust and hip circumference	Healthy overweight subjects	Parallel	Unclear	^?^	^?^	^+^	^+^	^?^	^?^	^?^	^?^	^?^

Thom 2007 Norway	Body weight, body mass index	Slight to moderately overweight subjects	Parallel	12/18	^?^	^?^	^+^	^+^	^?^	^?^	^?^	−	−

Dellalibera 1998 France	Body weight, body mass index	Overweight volunteers	Parallel	Unclear	^?^	^?^	^+^	^+^	^?^	^?^	^?^	−	−

Abbreviation: ITT (intention-to-treat); M/F: Males/Females.

Symbols: *: Unpublished study, ^+^: Yes, −: No, ^?^: Unclear.

**Table 2:** Main results of included RCTs^1^.

Author Year	GCE specification	No. of participants randomised	Age in yrs; Sex: M/F	Body weight at baseline	Dosage of GCE	Treatment duration	Main results; reported as means with standard deviations	Adverse events	Control for lifestyle factors
Ayton Res. 2009 (unpublished)	CGA enriched green coffee	62	Not reported	76.65 ± 7.25 kg (GCE) 77.44 ± 12.93 kg (PLA)	180 mg daily	4 weeks	Weight loss was 1.35 ± 0.81 kg and 0.12 ± 0.27 kg for GCE and PLA respectively	Not reported	Normal lifestyle

Thom 2007	CGA enriched green coffee	30	Not reported 12/18	85.2 ± 4.5 kg (GCE) 84.3 ± 4.3 kg (PLA)	200 mg daily	12 weeks	Mean weight loss was 5.4 ± 0.6 kg (GCE) and 1.7 ± 0.9 kg (PLA). Mean fat loss was 3.6 ± 0.3% (GCE) and 0.7 ± 0.4% (PLA)	No adverse events	Regular diet, normal level of exercise

Dellalibera 2007	Green coffee extract	50	Range: 19–75	Not reported	200 mg daily	12 weeks	^2^ Mean weight loss was 4.97 ± 0.32 kg and 2.45 ± 0.37 kg for GCE and PLA, respectively	Not reported	Not reported

Abbreviation: PLA: placebo

^1^ Unless otherwise specified, values are reported as means with standard deviations.

^2^ Values reported as means with standard errors.
